# Prognostic Significance of Tumor-Associated Macrophages in Solid Tumor: A Meta-Analysis of the Literature

**DOI:** 10.1371/journal.pone.0050946

**Published:** 2012-12-28

**Authors:** Qiong-wen Zhang, Lei Liu, Chang-yang Gong, Hua-shan Shi, Yun-hui Zeng, Xiao-ze Wang, Yu-wei Zhao, Yu-quan Wei

**Affiliations:** Department of Medical Oncology, Cancer Center, State Key Laboratory of Biotherapy, West China Hospital, Sichuan University, Chengdu, P. R. China; Johns Hopkins University, United States of America

## Abstract

**Purpose:**

Tumor associated macrophages (TAMs) are considered with the capacity to have both negative and positive effects on tumor growth. The prognostic value of TAM for survival in patients with solid tumor remains controversial.

**Experimental Design:**

We conducted a meta-analysis of 55 studies (n = 8,692 patients) that evaluated the correlation between TAM (detected by immunohistochemistry) and clinical staging, overall survival (OS) and disease free survival (DFS). The impact of M1 and M2 type TAM (n = 5) on survival was also examined.

**Results:**

High density of TAM was significantly associated with late clinical staging in patients with breast cancer [risk ratio (RR)  = 1.20 (95% confidence interval (CI), 1.14–1.28)] and bladder cancer [RR = 3.30 (95%CI, 1.56–6.96)] and with early clinical staging in patients with ovarian cancer [RR = 0.52 (95%CI, 0.35–0.77)]. Negative effects of TAM on OS was shown in patients with gastric cancer [RR = 1.64 (95%CI, 1.24–2.16)], breast cancer [RR = 8.62 (95%CI, 3.10–23.95)], bladder cancer [RR = 5.00 (95%CI, 1.98–12.63)], ovarian cancer [RR = 2.55 (95%CI, 1.60–4.06)], oral cancer [RR = 2.03 (95%CI, 1.47–2.80)] and thyroid cancer [RR = 2.72 (95%CI, 1.26–5.86)],and positive effects was displayed in patients with colorectal cancer [RR = 0.64 (95%CI, 0.43–0.96)]. No significant effect was showed between TAM and DFS. There was also no significant effect of two phenotypes of TAM on survival.

**Conclusions:**

Although some modest bias cannot be excluded, high density of TAM seems to be associated with worse OS in patients with gastric cancer, urogenital cancer and head and neck cancer, with better OS in patients with colorectal cancer.

## Introduction

Macrophages are a population of innate myeloid cells that are released from bone marrow as immature monocytic precursors and, after circulating in the blood stream, migrate into different tissues to undergo specific differentiation depending on local cues in the tissue [1], [2]. In response to different environment stimuli, macrophages can appear a range of different phenotypes [3]. The extremes of this range are recognized; the classically activated type M1 phenotype and the alternative activated M2 phenotype. The M1 macrophages are thought to be induced by interferon-γ, with or without lipopolysaccharide, tumor necrosis factor (TNF)-α, and activate cells of the adaptive immune system [4]. Differentiation of the M2 macrophages is induced by IL-4 or IL-13 and associated with parasite clearance, wound healing and dampen immune responses [5].

In 1863, it was fist found that a major leukocyte population was present in tumor, the so-called tumor-associated macrophages (TAM), which reflect the onset of cancer at site of previous chronic inflammation [1], [6]. These macrophages can induce neoplastic cell (cytotoxicity, apoptosis) and/or elicit tumor destructive reactions with the capacity to display both negative and positive effects on tumor growth depending on environmental stimuli of the tumor tissue [7], [8].

For long a large number of studies have been focused on identifying the prognostic value of TAM in solid tumors and most studies suggest that TAM is beneficial for tumor growth and, therefore, associated with poor prognosis [1]. However, there are some exceptions with high density of macrophages correlating with increased survival in different tumors [9]–[18] and even this contradiction has come up in the one type of tumor [11], [19]. This meta-analysis focused on the identifying diverse roles and functions of TAM and subpopulations of TAM for clinical outcome in patients with solid tumors.

## Materials and Methods

### Identification and Eligibility of Relevant Studies

We performed our meta-analysis according to a predetermined written protocol. To be eligible for our meta-analysis, studies had to deal with solid tumor at inclusion, to evaluate the correlation between TAM and survival, and to be published in English or Chinese languages. A computer-aided literature search of Pubmed (MEDLINE) 1950-present and EMBASE was conduced by combing search terms “cancer”, ”tumor”, “neoplasm”, “carcinoma”, “tumor-associated macrophage”, “tumor-infiltrating macrophage” and “intratumoral macrophage.” The deadline of the included articles was April 20^th^, 2012. Reference list from primary identified studies were also searched to prevent missing any studies by the electronic search strategies.

Inclusion criteria for primary studies were as follows: (1) proven diagnosis of solid tumor in humans, (2) using immunochemistry method to evaluate TAM by anti-CD68, M1-type TAM by anti-HLA-DR and M2-type TAM by anti-CD163, and (3) correlation of TAM with TNM staging, OS or DFS. Two independent reviewers processed primary assessment by identifying the eligibility of abstracts from database. Full articles were retrieved for further assessment if the eligibility was unclear from the abstracts. Any disagreements were resolved by serious discussion. We carefully examined the names of all authors and the medical centers involved in each publication to avoid duplication data. Whenever studies pertained to overlapping patients, we retained the studies with highest number of patients.

### Definitions and Standardizations

We used preconcerted rules to standardize as much as possible the definition of TAM positivity. As 20% was the used as a cutoff value in majority of the included studies [18], [20]–[26], we defined TAM positivity as positive cell stain in at least 20% of tumor cells. When different definitions were used, we contacted the primary author of each articles to retrieve the cutoff value they used. When cutoff value was not possible to retrieve, we accepted the cutoff was closet to the 20% cutoff level. When cutoff value was closed to 20%, which ranged from 16.3% to 25%, we also accepted the cutoff as 20%.

### Data Extraction

Data were carefully extracted from all of the included studies in duplicate by two of us, using a standard information collection form, with the following items, first author, year of publication, study design, median follow-up time, country of origin, number of patients involved, number of men included, mean or median age, tumor location, histological type, tumor-node-metastasis (TNM) staging, blinded reading, definition of TAM high, anti-cancer treatment(s) during follow up, OS or DFS or both. The main outcomes were tabulated in 2×2 tables showing the TNM staging status, occurrence or not of death or disease during follow-up according to TAM results.

### Statistical Analyses

Included studies were divided into three groups for analysis: those with data regarding TNM staging, those regarding OS and those regarding DFS. A study was considered significant when the P for comparing survival distribution between groups with high and low TAM was inferior to 0.05. A study was termed “positive” when a high TAM predicted a late clinical staging or poorer survival, “negative” when a high TAM predicted a early clinical staging or better survival, “indeterminate” when no significant relationship between TAM and clinical staging or survival was found.

For the quantitative aggregation of survival result, impacts of TAM on survival were reported for individual studies by estimating RRs with 95% confidence interval values. We first simply extracted RR and their 95%CI from the original article. If not available, the published data including number of patients at risk and total number of events in each groups from articles were used to estimate RR according to the methods described by Parmer et al [27]. When data were only available in the form of figures, we read Kaplan-Meier curves by Engauge Digitizer version 4.1 (free software down-loaded from http://sourceforge.net) and extracted survival data to reconstruct RRs and its 95%CI. An observed RR>1 indicated worse outcome for the TAM high group relative to TAM low group and would be considered statistically significant if the 95%CI did not overlap 1, with p<0.05. Sensitivity analyses were performed to examine the TAM effect of limiting the evaluation to studies using the 20% cutoff on prognosis. The effect of publication bias on the outcomes was assessed graphically using funnel plots, and funnel plot asymmetry was assessed by Egger's linear regression method. (p<0.05 was considered statistically significant publication bias) [28]. Meta-analyses were carried out by the Stata version 11.0 (Stata Corporation, College Station, TX, USA).

## Results

### Studies Selection and Characteristics

**Figure 1:**
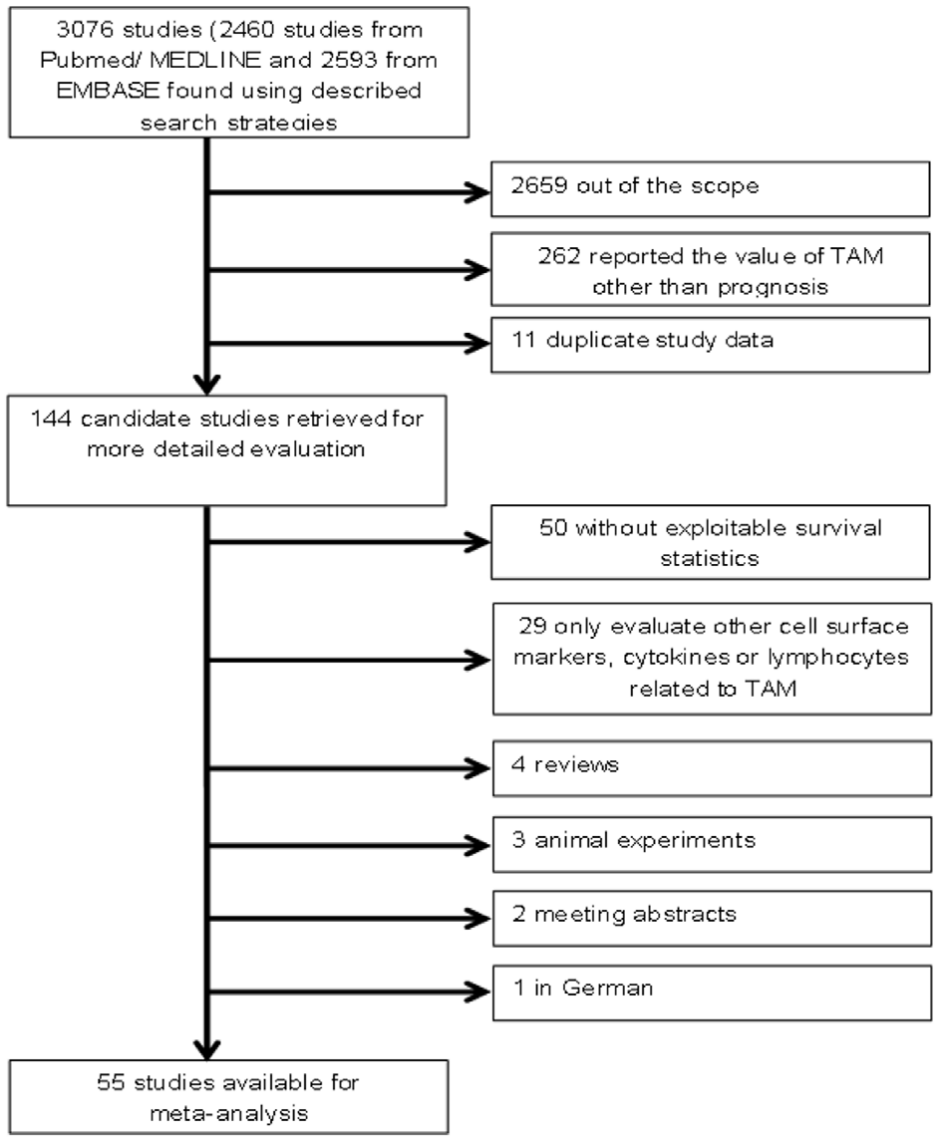
Flow chart of the literature search and selection of included studies.

The initial search algorithm retrieved a total of 3076 references and we evaluated 144 candidate studies in full text. Upon further review, 50 articles were eliminated due to inadequate data for meta-analysis and another 29 articles were out of scope because of evaluating other factors related to TAM (Figure 1). Overall, we identified 55 articles (n = 8693) with TAM measurements in patients with solid tumors.

The characteristics of included studies are listed in Table 1. The median sample size for all studies was 158 patients (range = 24–1902). The median sample size for staging was 189 patients, that for OS was 164 patients and that for DFS was 202 patients. The total proportion of male subjects was around 45% and that of patients in grade I/II was 43%. All evaluated IHC staining in formalin-fixed paraffin-embedded tissue blocks. The study design was more often a retrospective (N = 25) than a prospective cohort study (N = 5). Only 20% studies had performed blinded reading during evaluating TAM. The median follow-up time for all included studies ranged from 1.84 to 25 years. Of the 55 studies, cut-off value for definition of TAM high only could be retrieved from 32 original articles or by contacting authors.

**Table 1:** Characteristics of the eligible studies.

First author (ref)	Year	Study design	n (M/F)	Tumor location	Tumor stage I/II (III/IV)	Median follow-up (y)	Blinded reading	Staining for TAM high	RR estimation	Analysis	Result
**Gastrointestinal cancer (N = 19)**											
Khorana (29)	2003	Prosp.	131 (69/53)	131 Colon	11 (118)	5	NR	>2%	Data extrapolated, Survival curve	Stag., OS	Indeterminate
Tan (12)	2005	Retro.	60 (35/25)	60 Colon	26 (34)	NR	NR	>25%	Data extrapolated, Survival curve	Stag., OS	Negative
Bacman (30)	2007	NR	310 (189/121)	310 Colon	178 (132)	7.5	NR	NR	Data extrapolated	OS	Indeterminate
Forssell (15)	2007	Retro.	488 (271/217)	488 Colon	207 (254)	NR	NR	NR	Data extrapolated, Reported in text	Stag., OS	Indeterminate, negative
Zhou (18)	2010	NR	160 (94/66)	160 Colon	NR	5	Yes	20%	Data extrapolated, reported in text	Stag., OS	Negative
Ishigami (31)	2003	Retro.	97 (72/25)	97 Stom	67 (30)	NR	NR	>200 / HPF	Data extrapolated, Survival curve	Stag., OS	Indeterminate, positive
Ohno (10)	2005	Retro.	84 (57/27)	84 Stom	41 (43)	NR	Yes	>4.7%	Survival curve	DFS	Negative
Hass (32)	2009	Prosp.	52 (40/12)	50 Stom	39 (13)	5.9	NR	NR	Reported in text	DFS	Indeterminate
Kawahara (33)	2010	NR	111 (77/34)	111 Stom	36 (75)	NR	NR	NR	Reported in text	OS	Positive
Osinsky (34)	2011	Prosp.	105 (71/34)	105 stom	48 (57)	NR	NR	>23.0%	Reported in text	OS	Positive
Zhu (22)	2008	Retro.	105 (96/9)	105 Liver	86 (19)	1.84	NR	>20%	Data extrapolated, reported in text	Stag., OS, DFS	Indeterminate, positive
Li (24)	2009	Retro.	302 (260/42)	302 Liver	237 (65)	4.83	NR	>20%	Data extrapolated, reported in text	Stag., OS, DFS	Indeterminate, negative
Ding (35)	2009	Retro.	107 (87/20)	107 Liver	98 (39)	2.5	MR	>23%	Reported in text	OS, DFS	Positive
Kuang (36)	2009	Retro.	262 (NR)	206 Liver	249 (13)	NR	NR	NR	Reported in text	OS, DFS	Positive
Ju (23)	2009	Retro.	130 (112/18)	130 Liver	66 (64)	2.65	NR	>20%	Reported in text	Stag., OS, DFS	Positive
Koide (9)	2002	Retro.	56 (42/14)	56 Esophagus	30 (26)	NR	NR	NR	Data extrapolated, survival cure	Stag., OS	Indeterminate, negative
Guo (19)	2007	NR	137 (103/34)	137 Esophagus	86 (51)	NR	NR	>25%	Reported in text	Stag., OS	Indeterminate, positive
Kurahara (37)	2012	Retro.	76 (52/24)	52 Pancreas	29 (47)	NR	Yes	NR	Survival curve	OS, OS^b^	Indeterminate
Hasita (38)	2010	NR	39 (27/12)	55 Bile duct	29 (10)	3.33	Yes	NR	Data extrapolated, survival curve	Stag., OS, DFS, Stag.^b^, OS^b^, DFS^b^	Indeterminate
**Urogenital cancer (N = 20)**											
Leek (39)	1996	Retro.	101 (0/101)	5 Breast	NR	NR	NR	MD > = 12	Reported in text	OS, DFS	Positive
Toi (40)	1999	NR	229 (0/229)	229 Breast	NR	4	Yes	NR	Survival curve	DFS	Indeterminate
Valkovic (41)	2002	NR	97 (0/97)	97 Breast	82 (15)	NR	NR	NR	Data extrapolated	Stag.	Indeterminate
Bolat (42)	2006	Retro.	78 (0/78)	78 Breast	38 (10)	NR	NR	NR	Data extrapolated	Stag.	Indeterminate
Mahmound (43)	2012	Retro.	1902 (0/1902)	1902 Breast	220 (1682)	NR	NR	NR	Data extrapolated, survival curve	Stag., DFS	Positive, indeterminate
Salvesen (44)	1999	Prosp.	60 (0/60)	60 Endometrium	50(10)	11	NR	NR	Data extrapolated, reported in text	Stag., OS	Positive, indeterminate
Hashimoto (45)	2000	NR	109 (0/109)	109 Endometrium	56(53)	4.58	NR	NR	Data extrapolated	Stag., DFS	Indeterminate
Ohno (11)	2004	Retro.	70 (0/70)	70 Endometrium	NR	NR	NR	MD >10.7	Survival curve	DFS	Negative
Soeda (46)	2008	Retro.	76 (0/76)	76 Endometrium	59(17)	6.83	NR	NR	Survival curve	OS, DFS	Positive, indeterminate
Espinosa (47)	2010	NR	64 (0/64)	64 Endometrium	23(26)	NR	NR	NR	Data extrapolated	Stag.^b^	Indeterminate
Lissbrant (48)	2000	Retro.	85 (85/0)	85 Prostate	75(10)	NR	NR	> = 0.97%	Survival curve	Stag., OS	Indeterminate
Shimura (49)	2000	NR	81 (81/0)	81 Prostate	67(13)	3.9	NR	NR	Reported in text	DFS	Indeterminate
Nonomura (50)	2011	NR	131 (131/0)	131 Prostate	30(41)	2.9	NR	MD > = 22	Survival curve	DFS	Positive
Heller (51)	2002	Retro.	24 (0/24)	24 Cervix	20/4	NR	NR	NR	Data extrapolated	Stag.	Indeterminate
Kawanaka (52)	2008	Retro	73 (0/73)	73 Cervix	22/51	NR	Yes	MD > = 55	Survival curve	DFS	Indeterminate
Hanada (53)	2000	NR	63 (51/12)	63 Bladder	42/21	5.4	Yes	MD > = 67	Data extrapolated, reported in text	Stag., OS	Positive
Takayama (54)	2009	NR	41 (38/3)	41 Bladder	NR	25	NR	> = 4	Reported in text	DFS	Positive
Tanaka (55)	2004	NR	89 (0/89)	89 Ovary	22/67	6.8	NR	> = 25%	Data extrapolated, reported in text, survival curve	Stag., OS, DFS	Negative, positive, indeterminate
Wan (25)	2009	NR	67 (0/67)	67 Ovary	0/67	NR	NR	>20%	Survival curve	OS	Positive
Chai (20)	2008	NR	99 (42/57)	99 Uroth	NR	NR	NR	>20%	Reported in text	OS, DFS	Indeterminate, positive
**Lung cancer (N = 10)**											
Takanami (56)	1999	Retro.	113 (66/47)	113 Lung	61/52	NR	NR	MD>32	Survival curve	OS	Positive
Chen (57)	2005	NR	41 (27/14)	41 Lung	23/18	NR	NR	MD> = 163	Survival curve	DFS	Indeterminate
Welsh (13)	2005	Retro.	175 (116/59)	175 Lung	123/38	NR	Yes	NR	Data extrapolated	Stag., DFS	Negative
Zeni (58)	2007	NR	50 (43/7)	43 Lung	NR	NR	NR	>16.3%	Reported in text, survival curve	Stag., OS	Positive
Kawai (16)	2008	NR	199 (139/60)	199 Lung	NR	NR	NR	NR	Reported in text	OS	Negative
Ohri (17)	2009	Retro.	40 (16/24)	40 Lung	34/6	NR	NR	NR	Survival curve	OS	Negative
Al-shibli (59)	2009	Retro.	371 (253/82)	371 Lung	303/32	8	NR	> = 25%	Data extrapolated, survival curve	OS, DFS	Indeterminate
Ma (26)	2010	NR	50 (40/10)	50 Lung	33/17	NR	NR	>20%	Reported in text	OS^a^, OS^b^	Indeterminate
Ohtaki (60)	2010	Prosp.	170 (85/85)	170 Lung	NR	10.1	NR	NR	Data extrapolated	Stag., OS	Positive
Zhang (61)	2011	Retro.	65 (38/27)	65 Lung	38/27	NR	Yes	TAM counts >102	Survival curve	OS	Positive
**Head and neck cancer (N = 4)**											
Liu (21)	2008	NR	112 (93/19)	112 Oral	50/62	NR	NR	>20%	Data extrapolated, survival curve	Stag., OS	Positive
Fujii (62)	2012	NR	108 (31/10)	108 Oral	46/62	NR	NR	> = 2/HPF	Data extrapolated, reported in text	Stag., stag.^b^, OS^b^	Positive, indeterminate, positive
Peng (14)	2006	NR	60 (38/22)	60 Oral	15/45	NR	NR	> = 63.7/HPF	Data extrapolated	OS	Negative
Lin (63)	2011	NR	84 (77/7)	84 Lar	30/54	NR	NR	> = 10/HPF	Data extrapolated, reported in text	Stag., OS, DFS	Indeterminate, indeterminate, positive
**Thyroid cancer (N = 1)**											
Ryder (64)	2008	NR	37 (13/24)	37 Thyr	5/32	NR	Yes	> = 10/HPF	Survival curve	OS	Positive
**Mesothelioma (N = 1)**											
Burt (65)	2011	Retro.	667 (531/136)	667 Meso	64/603	NR	Yes	NR	Reported in text	OS	Indeterminate

Of the included studies, 19 studies focused on gastrointestinal cancers, including colorectal cancer (N = 5), gastric cancer (N = 5), liver cancer (N = 5), esophagus cancer (N = 2), pancreatic caner (N = 1) and cholangiocarcinoma (N = 1). 20 studies analyzed the impact of TAM on survival in patients with urogenital cancers, including breast cancer (N = 5), endometrial cancer (N = 5), prostate cancer (N = 3), cervical cancer (N = 2), bladder cancer (N = 2), ovary cancer (N = 2) and urothelial cancer (N = 1). 5 studies mentioned the value of M2-type TAM on survival [26], [37], [38], [47], [62].

### Data Synthesis: Clinical Staging

**Figure 2:**
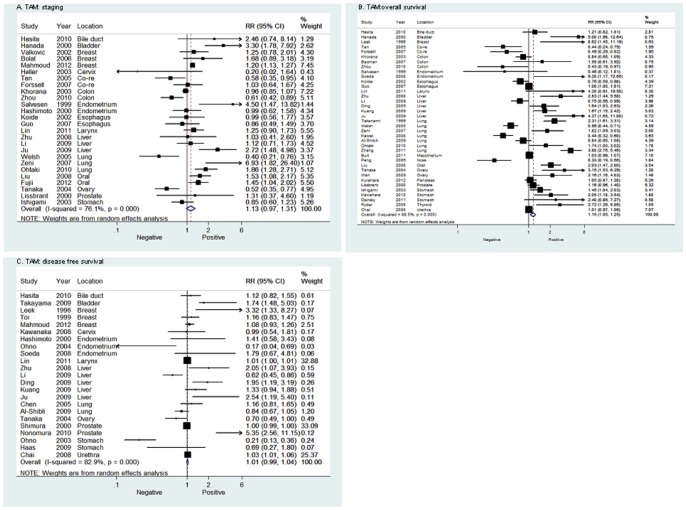
Forrest plots and meta-analysis of studies evaluating RR of high TAM counts as compared to low counts. Clinical staging and survival data are reported as (**A**) staging, (**B**) overall survival (OS) and (**C**) disease free survival (DFS).

As shown in Figure 2A, the combining data of TAM on clinical staging showed a nonstatistically significant RR of 1.13 (95%CI 0.97–1.31). For sub-group analysis, all of the studies were dispatched into several classes according to the tumor location. Our data showed that high density of TAM was significantly higher in patients with advanced tumor stage (III+IV) than in the patients with early stage (I+II), which occurred in breast cancer [RR = 1.20 (95%CI, 1.14–1.28)], oral cancer [RR = 1.49 (95%CI, 1.17–1.89)] and bladder cancer [RR = 3.30 (95%CI, 1.78–7.92)],whereas 1 article focused on ovary cancer found high density of TAM was associated with early stage [RR = 0.52 (95%CI, 0.36–0.77)]. Other sub-group analysis found no relation between TAM and clinical staging. In studies specific defining TAM positivity as at least 20% positive staining cells in tumor tissue, no significant relation was found between TAM and clinical staging (RR = 1.23 (95%CI, 0.74–2.02). Analysis for M2-type TAM on staging also showed no significant effect [RR = 1.43 (95%CI, 0.70–2.93)].

### Data Synthesis: Overall Survival

**Figure 3:**
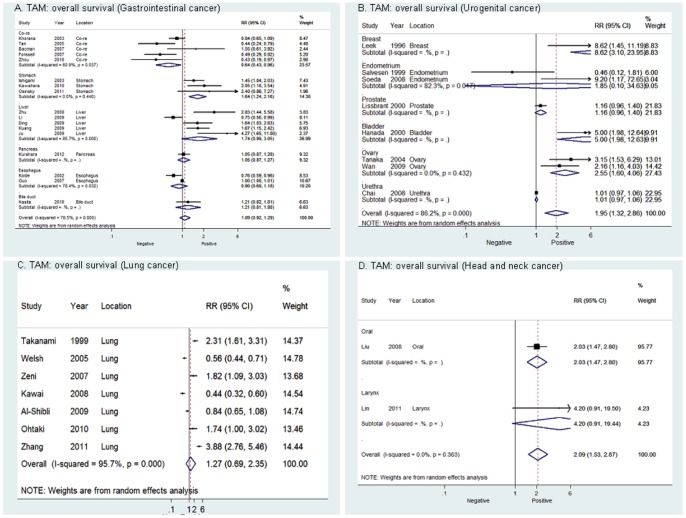
Forrest plots and meta-analysis of studies evaluating RR of high TAM counts as compared to low counts in different subgroup of tumors. Clinical staging and survival data are reported as (**A**) gastrointestinal cancer, (**B**) urogenital cancer, (**C**) lung cancer and (**D**) head and neck cancer.

For overall population, high density of TAM was associated with a worse prognosis regarding overall survival (Figure 2B). However, mortality was only 1.15-fold higher in high TAM patients with solid tumor, which showed modest effect. In sub-group analysis (Figure 3), high density of TAM was significantly correlated with poor OS in patients with urogenital cancer [RR = 1.95 (95%CI, 1.32–2.86)], including breast cancer [RR = 8.62 (95%CI, 3.10–23.95)], endometrial cancer [RR = 1.85 (95%CI, 0.10–34.63)], prostate cancer [RR = 1.16 (95%CI, 0.96–1.40)], bladder cancer [RR = 5.00 (95%CI, 1.98–12.63)], ovary cancer [RR = 2.55 (95%CI, 1.60–4.06)] and urothelial cancer [RR = 1.01 (95%CI, 1.32–2.86)]. In addition, gastric cancer and oral cancer showed significant RR between TAM and OS [RR = 1.54 (95%CI 1.24–2.16), 2.03 (95%CI 1.47–2.80)]. However, analysis of studies on colorectal cancer showed that there was a significant correlation between high density of TAM and longer OS [RR = 0.64 (95%CI, 0.43–0.96)]. No significant correlation between TAM and OS was found in other sub-group analysis. Five studies reported that there was no significant correlation between M2-type TAM and OS [RR = 0.98 (95%CI, 0.71–1.35)], one study reported that the M1-type TAM density in the tumor islet is positively associated with extended survival in patients with lung cancer [17]. There was also no difference in the summary estimate of TAM on overall survival when cutoff value was specific to 20% (RR = 1.41 (95%CI, 1.03–2.09).

### Data Synthesis: Disease free survival

For the overall population, no significant relation was observed between TAM and DFS [RR = 1.01 (95%CI, 0.99–1.04)](Figure 2C). No significant effect was showed in sub-group analysis. One article [38] with data on M2-type TAM and DFS also showed a non-significant effect [RR = 1.09 (95%CI, 0.77–1.54)]. Analysis for specific 20% cutoff also showed no significant effect (RR = 1.19 (95%CI, 0.78–1.57).

### Data Synthesis in sub-groups: clinic-pathological factors

The impact of TAM density on clinic-pathological in patients with different cancer was further analyzed and described in Table 2. Two studies [12], [29] showed a significant correlation between T status and TAM density, nevertheless, no correlation was found between TAM density and lymph node metastasis nor distant metastasis. High density of TAM was also correlated with nonmucinous type of colon cancer. Additionally, two of the studies on gastric cancer described that low density of TAM was significantly correlated with lymph node metastasis [31], [32]. In the breast cancer group, Negative effects of TAM were found not only on TNM stage, but also histological grade, lymph node metastasis, tumor size, vascular invasion and HER-2 status. Similar phenomena were seen in bladder cancer and oral squamous group, and high density of TAM was significantly correlated with TNM stage, T status, lymph node metastasis and distant metastasis. In addition, one study demonstrated that the density of TAM was significantly lower in patients with advanced tumor stage (III/IV) than in patients with early stage (I/II) [55].

**Table 2:** Meta-analysis of the subgroups based on clinic-pathological factors related to TAM density from the available published studies.

Cancer type	Clinic-pathological factors	Number of studies	Number of total patients	HR (95% CI)	*p*-value	Results	Reference
**Colon cancer**							
	TNM Stage (I/II vs. III/IV)	2	619	0.71 (0.35, 1.45)	0.194	Indeterminate	(15), (29)
	T status (T1/T2 vs. T3/T4)	2	191	0.35 (0.13, 0.90)	0.246	Positive	(12), (29)
	Lymph node metastasis	2	370	0.72 (0.13, 4.06)	0.013	Indeterminate	(12), (30)
	Distant metastasis	3	530	0.72 (0.24, 2.11)	0.005	Indeterminate	(12), (18), (30)
	Histological grade (well/moderate vs. poor)	3	858	0.26 (0.06, 1.06)	0.005	Indeterminate	(12), (15), (30)
	Pathologic classification (mucinous vs. nonmucinous)	2	648	2.53 (1.35, 4.73)	0.675	Negative	(15), (18)
**Gastric cancer**							
	TNM Stage (I/II vs. III/IV)	1	97	2.40 (0.98, 5.91)	NR	Indeterminate	(31)
	T status (T1/T2 vs. T3/T4)	1	97	1.36 (0.54, 3.46)	NR	Indeterminate	(31)
	Lymph node metastasis	2	149	0.39 (0.19, 0.80)	0.302	Positive	(31), (32)
	Distant metastasis	1	52	1.30 (0.40, 4.28)	NR	Indeterminate	(32)
	Histological grade (well/moderate vs. poor)	1	97	0.54 (0.22, 1.32)	NR	Indeterminate	(31)
**Liver cancer**							
	TNM Stage (I/II vs. III/IV)	3	514	1.27 (0.84, 1.93)	0.702	Indeterminate	(22), (24), (35)
	Vascular invasion (absent vs. present)	3	514	1.38 (0.87, 2.21)	0.410	Indeterminate	(22), (24), (35)
	Tumor differentiation	2	409	1.08 (0.70, 1.66)	0.956	Indeterminate	(24), (35)
	Tumor size (≤5 vs. >5cm)	2	409	1.76 (1.19, 2.60)	0.329	Negative	(24), (35)
	Hepatitis history (No vs. Yes)	2	407	1.45 (0.80, 2.60)	0.456	Indeterminate	(22), (24)
	Liver cirrhosis (No vs. Yes)	3	514	1.18 (0.75, 1.88)	0.886	Indeterminate	(22), (24), (35)
	Fibrous capsule (absent vs. present)	3	514	0.83 (0.58, 1.19)	0.750	Indeterminate	(22), (24), (35)
**Esophagus cancer**							
	T status (T1/T2 vs. T3/T4)	1	56	2.73 (0.70, 10.60)	NR	Indeterminate	(9)
	Lymph node metastasis	1	56	0.75 (0.21, 2.59)	NR	Indeterminate	(9)
	Lymphatic invasion (absent vs. present)	1	56	3.21 (0.81, 12.8)	NR	Indeterminate	(9)
	Venous invasion (absent vs. present)	1	56	6.22 (1.48, 26.1)	NR	Negative	(9)
**Intrahepatic cholangiocarcinoma**							
	UICC stage (I/II vs. III/IV)	1	55	3.31 (0.71, 15.4)	NR	Indeterminate	(38)
	Histological grade (well/moderate vs. poor)	1	55	5.25 (0.93, 29.7)	NR	Indeterminate	(38)
	Lymph node metastasis	1	55	1.06 (0.19, 6.05)	NR	Indeterminate	(38)
	Vascular invasion (absent vs. present)	1	55	2.13 (0.34, 13.2)	NR	Indeterminate	(38)
	Tumor size (<4 vs. ≥4cm)	1	55	1.38 (0.39, 4.87)	NR	Indeterminate	(38)
**Breast cancer**							
	TNM Stage (I/II vs. III/IV)	1	78	1.20 (1.14, 1.28)	NR	Negative	(42)
	Histological grade (I/II vs. III)	3	2077	3.42 (2.71, 4.30)	0.742	Negative	(41), (42), (43)
	Lymph node metastasis	3	2077	1.29 (1.04, 1.62)	0.604	Negative	(41), (42), (43)
	Tumor size (≤2 vs. >2cm)	3	2077	1.43 (1.14, 1.80)	0.963	Negative	(41), (42), (43)
	Vascular invasion (absent vs. present)	1	1902	1.74 (1.35, 2.23)	NR	Negative	(43)
	HER-2 status (negative vs. positive)	1	1902	2.59 (1.75, 3.85)	NR	Negative	(43)
**Endometrial cancer**							
	FIGO stage (I/II vs. III/IV)	2	169	2.34 (0.36, 15.39)	0.021	Indeterminate	(44), (45)
	Lymph node metastasis	1	109	0.43 (0.12, 1.53)	NR	Indeterminate	(45)
	Myometrial invasion (negative vs. positive)	1	109	2.09 (0.65, 6.69)	NR	Indeterminate	(45)
	Histological grade (I/II vs. III)	2	169	4.34 (0.70, 27.08)	0.044	Indeterminate	(44), (45)
	Pathologic classification (Endometrioid vs.non-endometrioid)	1	109	1.37 (0.41, 4.62)	NR	Indeterminate	(45)
**Prostate cancer**							
	T status (T1/T2 vs. T3/T4)	1	85	3.30 (1.56, 6.96)	NR	Indeterminate	(48)
	Distant metastasis	1	85	1.06 (0.25, 4.54)	NR	Indeterminate	(48)
**Cervical cancer**							
	FIGO stage (I/II vs. III/IV)	2	97	0.68 (0.06, 8.26)	0.063	Indeterminate	(51), (52)
	Lymph node metastasis	1	24	1.75 (0.26, 11.7)	NR	Indeterminate	(51)
**Bladder cancer**							
	TNM Stage (I/II vs. III/IV)	1	63	5.76 (1.76, 18.9)	NR	Negative	(53)
	T status (T1/T2 vs. T3/T4)	1	63	17.6 (4,34, 71.1)	NR	Negative	(53)
	Distant metastasis	1	63	12.4 (2.50, 61.0)	NR	Negative	(53)
	Vascular invasion (absent vs. present)	1	63	10.8 (1.26, 92.4)	NR	Negative	(53)
**Ovary cancer**							
	TNM Stage (I/II vs. III/IV)	1	89	0.52 (0.35, 0.77)	NR	Positive	(54)
	Lymph node metastasis	1	89	0.14 (0.05, 0.35)	NR	Positive	(54)
	Tumor differentiation	1	89	0.79 (0.34, 1.83)	NR	Indeterminate	(54)
	Tumor size (≤5 vs. >5cm)	1	89	0.94 (0.41, 2.17)	NR	Indeterminate	(54)
	Histological type (serous vs. nonserous)	1	89	0.81 (0.34, 1.9)	NR	Indeterminate	(54)
**Lung cancer**							
	Pathologic stage (I vs. II/III/IV	2	345	1.29 (0.24, 6.78)	<0.001	Indeterminate	(13), (60)
	Tumor differentiation	1	170	5.80 (2.99, 11.2)	NR	Negative	(60)
	T status (T1/T2 vs. T3/T4)	1	170	2.70 (1.40, 5.21)	NR	Negative	(60)
	Lymph node metastasis	1	170	2.72 (1.27, 5.82)	NR	Negative	(60)
	Vessel invasion	1	170	3.24 (1.69, 6.24)	NR	Negative	(60)
**Oral squamous cell carcinoma**							
	TNM Stage (I/II vs. III/IV)	2	220	2.53 (1.46, 4.38)	0.888	Negative	(21), (62)
	T status (T1/T2 vs. T3/T4)	2	220	2.33 (1.34, 4.03)	0.328	Negative	(21), (62)
	Lymph node metastasis	2	220	2.56 (1.46, 4.47)	0.528	Negative	(21), (62)
	Tumor differentiation	2	220	1.32 (0.68, 2.57)	0.294	Indeterminate	(21), (62)
**Thyroid cancer**							
	TNM Stage (I/II vs. III/IV)	1	37	4.31 (0.42, 43.7)	NR	Indeterminate	(64)
	Distant metastasis	1	37	4.17 (0.66, 26.1)	NR	Indeterminate	(64)

### Evaluation of publication bias

**Figure 4:**
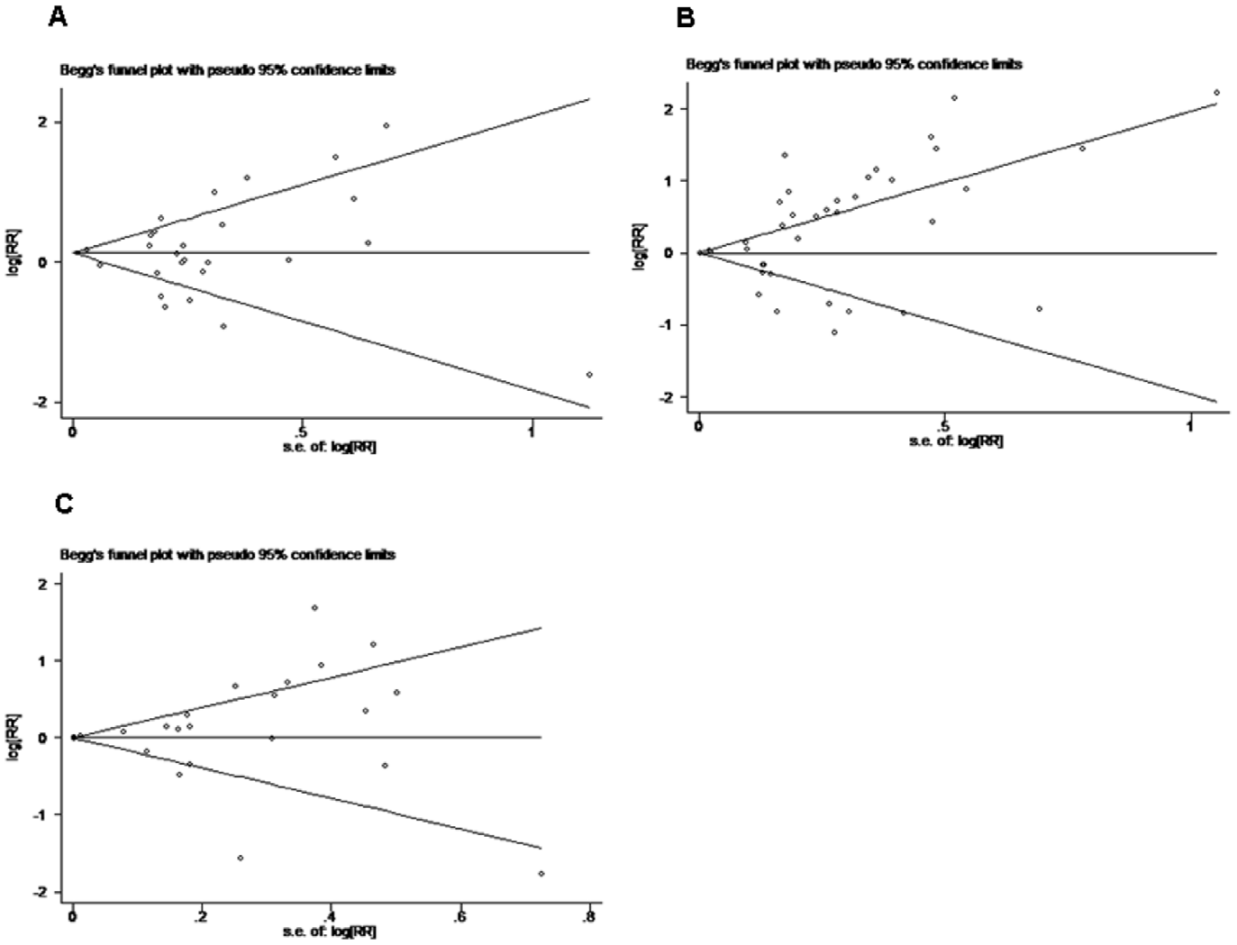
Funnel graph for assessment of potential publication bias in studies of TAM density in patients with solid tumor. (**A**) Staging, (**B**) OS, (**C**) DFS. The funnel graph plots log HR against the standard error of the log HR. The result of the Egger's test showed no statistical significant (p>0.05).

Both Begg's funnel plot and Egger's test were performed to assess the publication bias in all studies evaluating staging, OS, DFS separately, and evaluation was also performed in sub-group analysis. Begg's funnel plot did not reveal any evidence of significant asymmetry in the overall meta-analysis of staging (p = 0.679), OS (p = 0.065) and DFS (p = 0.792)(Figure 4). There was also no indication of publication in Egger's test of staging (p = 0.993), OS (p = 0.058) and DFS (p = 0.357). For sub-group evaluation of publication bias, no significant publication bias was shown from either Egger's or Begg's test (not shown).

## Discussion

So far, a group of original articles and reviews has studied the prognostic significance of TAM in solid tumors, and the presence of both significant and non-significant studies addressing the importance of TAM on survival made it necessary to perform a quantitative aggregation of the survival results. The present result showed that high density of TAM, as detected with immunohistochemistry, was significant associated with worse overall survival in solid tumor, with a global RR of 1.15. As potential bias exists between studies on different tumors, subgroup analysis was also performed, which suggested that high density of TAM was significant associated with OS in patients with gastric cancer [RR = 1.64 (95%CI, 1.24–2.16)], breast cancer [RR = 8.62 (95%CI, 3.10–23.95)], bladder caner [RR = 5.00 (95%CI, 1.98–12.63)], ovarian cancer [RR = 2.55 (95%CI, 1.60–4.06)], oral cancer [RR = 2.03 (95%CI, 1.47–2.80)] and thyroid cancer [RR = 2.72 (95%CI, 1.26–5.86)]. Moreover, there showed positive effect in patients with colorectal cancer [RR = 0.64 (95%CI, 0.43–0.96)]. However, no significant effect was seen between TAM and DFS.

When comparing the results of different types of tumors, several key differences were observed. As mentioned above, although macrophages under certain conditions can kill tumor cells, they can also play potential roles as tumor promoters to secrete a variety of factors that directly stimulate tumor invasion and metastasis. The combing effect of TAM on prognosis in patients with different tumors depends on stimulating factors from two opposite aspects in tumor environments. In this meta-analysis, we reach a conclusion that high TAM infiltration is associated with worsen prognosis in patients with urogenital cancer or gastric cancer, not all cancer type. In other hand, TAM showed antitumorigenic properties in combing 5 studies on colorectal cancer, resulting in improved prognosis.

To further investigate the prognostic value of TAM in different type of cancer, we analyzed the relation between the density of TAM and clinic-pathological factors that was also associated with outcome of cancer patients. As the density of TAM has a negative effect on survival in patients with gastric cancer, breast cancer, bladder cancer, ovary cancer, and oral squamous cell carcinoma, negative effects are also seen in clinic-pathological factors such as TNM staging (breast cancer, bladder cancer and oral squamous cell carcinoma), T status (breast cancer and oral squamous cell carcinoma), lymph node metastasis (breast cancer, bladder cancer and oral squamous cell carcinoma), and distant metastasis (bladder cancer), which contributed to tumor progression and patient survival. Interestingly, there also demonstrated a positive effect of TAM on lymph node metastasis in gastric and ovary cancer, which indicated that high density of TAM was associated with less probability of lymph node metastasis, however, significant negative effect was shown on overall survival. Thus, more studies are needed to clarify this ambivalent phenomenon. Contrary to tumors we mentioned above, our data suggested that an incremental increase in density of TAM improved overall survival in patients with colon cancer, with a homodromous effect on T status. There was also a trend towards lower rate of lymph node metastasis and distant metastasis in TAM rich tumors. In addition, a high density of TAM infiltration was found related to nonmucious type of colon cancer.

The mechanisms behind the oncogenic and anti-tumorigenic effects of TAMs have not been fully elucidated and a great number of studies have focused on explaining these apparently contradictory effects of TAM in different cancer outcome. The functions of TAM in different type of tumors are concerned as the most important determining factor to the prognosis, which are profoundly affected by microenvironmental signals and can range from powerful stimulation of inflammatory responses to induction of immunosuppression [66]. Tumor necrosis factor (TNF)-α, nitric oxide (NO), and monocyte chemoattractant protein (MCP)-1 released from TAM are major intermediate molecules for tumor cell killing [67]–[69], and TAM associated vascular endothelial growth factor (VEGF) and matrix metalloproteinases (MMPs) are independent predictor of poor prognostic factor in cancer patients [34]. In addition, a macrophage balance hypothesis between M1 and M2 type macrophages has been proposed and two different macrophage populations range from polarized potent killer M1 cells to alternatively activated M2 macrophages with tumor-promoting capability [70]. However, this study showed no significant relation between the density of two phenotype of TAM and survival of patients. Furthermore, histological classification of the tumor should also be considered as a factor correlated with the function of TAM. A previous study on colon cancer demonstrated that a histologically more malignant phenotype was associated with macrophage infiltration, disorganized matrix deposition, and extensive stromal reaction [71]. In one included study of this meta-analysis, the number of infiltrating TAM was found to be significantly correlated with poor outcome in patients with intestinal type of gastric cancer, but not in patients with diffuse type, indicating that TAM could affect malignant progression and prognosis on the basis of the histological type of gastric cancer [33].

Although the results of meta-analysis are considered as gold standards by authors worldwide [72], potential bias still exists between studies and cannot be completely eliminated. Although Begg's funnel plot and Egger's test were performed in this meta-analysis and found no statistically significant publication bias, results of this study should be interpreted very cautiously and several aspects of importance in this field should be discussed. First of all, we only included studies from which we could extract RR or estimate RR, leading to data inaccessible for data aggregation from studies, which only showed the conclusion on this topic without data presented. Take one excluded study on evaluating the prognostic value of TAM on oral cavity and oropharyngeal squamous cell carcinoma for example, they found that macrophage content was an independent predictor of lymph node metastasis, however, no data was accessible for meta-analysis from this study [73]. Furthermore, considerable attention should be paid to some cancers with few study included in this meta-analysis study. TAM was found associated with worse prognosis in one study on oral cancer [RR = 2.03 (95%CI, 1.47–2.80)] [21] and one study on thyroid cancer [RR = 2.72 (95%CI, 1.26–5.86)] [64]. As meta-analysis could not be performed with such small number of primary studies, more researches are needed in further investigation on these tumors.

Second, macrophages can be identified by cell surface markers, expression of transcriptional factors, the production of cytokines and their functions in vitro [2]. However, we only included literatures evaluating TAM with the use of antibodies to the glycoprotein CD68. Sikert et al quantified TAM by immunohistochemistry with antibodies to PG-M1, KP-1, MRP8, MRP14 and MRP8/14 antigens and found different TAM subpopulation were positively correlated with clinicopathological characteristics in colon cancer [74]. Macrophage differentiation, growth, and chemotaxis are regulated by several growth factors, including colony-stimulating factor (CSF)-1, macrophage chemoattractant protein (MCP)-1 and extracellular matrix protease such as urokinase-type plasminogen activator (uPA) [75]. For example, over expression of CSF is associated with poor prognosis in nongynecological leiomyosarcoma [76]. In breast cancer, TAM density is showed correlated with angiogenesis and poor prognosis [77]–[79]. Ohba et al provide the evidence that uPA has prognostic value in patients with renal cell carcinoma via TAM [80]. Also other factors could be used to evaluate M1 and M2 type macrophages in tumor tissues. As M2 type TAM express high level of interleukin-10 (IL-10) which can be used to discriminate between M1 and M2 macrophages [81], [82], a study assessed IL-10 expression in TAM, and found the high level of IL-10 in TAM significantly correlated with clinical staging and histologic poor differentiation in patients with lung cancer [83]. So, considerable attention should be paid to various kinds of factors related to density of TAM, which might be a potential prognostic marker in solid tumor.

Third, variability in definitions, outcomes, measurements, experimental procedure, and even antibody concentration may contribute to heterogeneity between studies [84]. Multivariate analyses was tried to minimize confounding bias, but the factors controlled for were few and differed between studies. Quality criteria are needed for future studies in this field, and we make the following recommendation: blindly assess the prognostic marker to patient outcome, adequately describe the assay method used for TAM evaluation including antibody concentration and cut-off value staining for TAM high, and precisely define outcome with certain follow-up time. More importantly, in this meta-analysis, some studies have used 20% as the cutoff value, whereas others have chosen score system, mean, median or arbitrary cutoff values, thus cutoff value is a source of considerable interstudy heterogeneity. Although specific synthesis of studies using standardized cutoff value on survival did not differ significantly form the overall result in the total population analysis, conclusions need to be considered cautiously.

In conclusion, it is clear that TAM has protumorigenic as well as antitumorigenic properties in solid tumor. As discussed above, there have been showed a “macrophage balance” on prognosis depending on the microenvironment of the tumor tissue in different type of solid tumor. It may be possible in the future to use or induce activated macrophages to restrain tumor growth and improve patient survival, through altering tumor microenvironment. Moreover, targeted therapies that uniquely strike macrophages may provide innovative therapeutic strategies against tumor progression.

## References

[1] Bingle L, Brown NJ, Lewis CE (2002) The role of tumour-associated macrophages in tumour progression: implications for new anticancer therapies. J Pathol 196: 254–265. 11857487 10.1002/path.1027

[2] Heusinkveld M, van der Burg SH (2011) Identification and manipulation of tumor associated macrophages in human cancers. J Transl Med 9: 216. 22176642 10.1186/1479-5876-9-216 PMC3286485

[3] Siveen KS, Kuttan G (2009) Role of macrophages in tumour progression. Immunol Lett 123: 97–102. 19428556 10.1016/j.imlet.2009.02.011

[4] Mantovani A, Sozzani S, Locati M, Allavena P, Sica A (2002) Macrophage polarization: tumor-associated macrophages as a paradigm for polarized M2 mononuclear phagocytes. Trends Immunol 23: 549–555. 12401408 10.1016/s1471-4906(02)02302-5

[5] Biswas SK, Mantovani A (2010) Macrophage plasticity and interaction with lymphocyte subsets: cancer as a paradigm. Nat Immunol 11: 889–896. 20856220 10.1038/ni.1937

[6] Virchow R (1863) Aetologie der neoplastichen Geschwulste/ Pathogenie der neoplastischen Geschwulste. Die Krankhaften Geschwulste Berlin: Verlag von August Hirshwald: 57–101.

[7] Mantovuni BB, Colotta F, Sozzani S, Ruco L (1992) The origin and function of tumor associated macrophages. Immunol Today 13: p. 2–74. 10.1016/0167-5699(92)90008-U 1388654

[8] Mantovuni A (1994) Biology of disease: tumor-associated macrophages in neoplastic progression: a paradigm for the in vivo function of chemokines. Lab Invest 71: 5–16. 7518882

[9] Koide N, Nishio A, Hiraguri M, Kishimoto K, Nakamura T, et al (2002) Differences and relationships of thymidine phosphorylase expression in tumor-associated macrophages and cancer cells in squamous cell carcinoma of the esophagus. Dis Esophagus 15: 67–73. 12060046 10.1046/j.1442-2050.2002.00223.x

[10] Ohno S, Inagawa H, Dhar DK, Fujii T, Ueda S, et al (2003) The degree of macrophage infiltration into the cancer cell nest is a significant predictor of survival in gastric cancer patients. Anticancer Res 23: 5015–5022. 14981961

[11] Ohno S, Ohno Y, Suzuki N, Kamei T, Koike K, et al (2004) Correlation of histological localization of tumor-associated macrophages with clinicopathological features in endometrial cancer. Anticancer Res 24: 3335–3342. 15515429

[12] Tan SY, Fan Y, Luo HS, Shen ZX, Guo Y, et al (2005) Prognostic significance of cell infiltrations of immunosurveillance in colorectal cancer. World J Gastroenterol 11: 1210–1214. 15754407 10.3748/wjg.v11.i8.1210 PMC4250716

[13] Welsh TJ, Green RH, Richardson D, Waller DA, O'Byrne KJ, et al (2005) Macrophage and mast-cell invasion of tumor cell islets confers a marked survival advantage in non-small-cell lung cancer. Journal of Clinical Oncology 23: 8959–8967. 16219934 10.1200/JCO.2005.01.4910

[14] Peng J, Ding T, Zheng LM, Shao JY (2006) Influence of tumor-associated macrophages on progression and prognosis of nasopharyngeal carcinoma. Ai Zheng 25: 1340–1345. 17094898

[15] Forssell J, Oberg A, Henriksson ML, Senling R, Jung A, et al (2007) High macrophage infiltration along the tumor front correlates with improved survival in colon cancer. Clin Cancer Res 13: 1472–1479. 17332291 10.1158/1078-0432.CCR-06-2073

[16] Kawai O, Ishii G, Kubota K, Murata Y, Naito Y, et al (2008) Predominant infiltration of macrophages and CD8(+) T Cells in cancer nests is a significant predictor of survival in stage IV nonsmall cell lung cancer. Cancer 113: 1387–1395. 18671239 10.1002/cncr.23712

[17] Ohri CM, Shikotra A, Green RH, Waller DA, Bradding P (2009) Macrophages within NSCLC tumour islets are predominantly of a cytotoxic M1 phenotype associated with extended survival. Eur Respir J 33: 118–126. 19118225 10.1183/09031936.00065708

[18] Zhou Q, Peng RQ, Wu XJ, Xia Q, Hou JH, et al (2010) The density of macrophages in the invasive front is inversely correlated to liver metastasis in colon cancer. J Transl Med 8: 13. 20141634 10.1186/1479-5876-8-13 PMC2841127

[19] Guo SJ, Lin DM, Li J, Liu RZ, Zhou CX, et al (2007) Tumor-associated macrophages and CD3-zeta expression of tumor-infiltrating lymphocytes in human esophageal squamous-cell carcinoma. Dis Esophagus 20: 107–116. 17439593 10.1111/j.1442-2050.2007.00655.x

[20] Chai CY, Chen WT, Hung WC, Kang WY, Huang YC, et al (2008) Hypoxia-inducible factor-1alpha expression correlates with focal macrophage infiltration, angiogenesis and unfavourable prognosis in urothelial carcinoma. J Clin Pathol 61: 658–664. 17908805 10.1136/jcp.2007.050666

[21] Liu SY, Chang LC, Pan LF, Hung YJ, Lee CH, et al (2008) Clinicopathologic significance of tumor cell-lined vessel and microenvironment in oral squamous cell carcinoma. Oral Oncol 44: 277–285. 17475541 10.1016/j.oraloncology.2007.02.007

[22] Zhu XD, Zhang JB, Zhuang PY, Zhu HG, Zhang W, et al (2008) High expression of macrophage colony-stimulating factor in peritumoral liver tissue is associated with poor survival after curative resection of hepatocellular carcinoma. J Clin Oncol 26: 2707–2716. 18509183 10.1200/JCO.2007.15.6521

[23] Ju MJ, Qiu SJ, Fan J, Xiao YS, Gao Q, et al (2009) Peritumoral activated hepatic stellate cells predict poor clinical outcome in hepatocellular carcinoma after curative resection. Am J Clin Pathol 131: 498–510. 19289585 10.1309/AJCP86PPBNGOHNNL

[24] Li YW, Qiu SJ, Fan J, Gao Q, Zhou J, et al (2009) Tumor-infiltrating macrophages can predict favorable prognosis in hepatocellular carcinoma after resection. J Cancer Res Clin Oncol 135: 439–449. 18781326 10.1007/s00432-008-0469-0 PMC12160305

[25] Wan T, Liu JH, Zheng LM, Cai MY, Ding T (2009) Prognostic significance of tumor-associated macrophage infiltration in advanced epithelial ovarian carcinoma. Ai Zheng 28: 323–327. 19619451

[26] Ma J, Liu L, Che G, Yu N, Dai F, et al (2010) The M1 form of tumor-associated macrophages in non-small cell lung cancer is positively associated with survival time. BMC Cancer 10: 112. 20338029 10.1186/1471-2407-10-112 PMC2851690

[27] Parmar MK, Torri V, Stewart L (1998) Extracting summary statistics to perform meta-analyses of the published literature for survival endpoints. Stat Med 17: 2815–2834. 9921604 10.1002/(sici)1097-0258(19981230)17:24<2815::aid-sim110>3.0.co;2-8

[28] Egger M, Davey Smith G, Schneider M, Minder C (1997) Bias in meta-analysis detected by a simple, graphical test. BMJ 315: 629–634. 9310563 10.1136/bmj.315.7109.629 PMC2127453

[29] Khorana AA, Ryan CK, Cox C, Eberly S, Sahasrabudhe DM (2003) Vascular endothelial growth factor, CD68, and epidermal growth factor receptor expression and survival in patients with Stage II and Stage III colon carcinoma: a role for the host response in prognosis. Cancer 97: 960–968. 12569594 10.1002/cncr.11152

[30] Bacman D, Merkel S, Croner R, Papadopoulos T, Brueckl W, et al (2007) TGF-beta receptor 2 downregulation in tumour-associated stroma worsens prognosis and high-grade tumours show more tumour-associated macrophages and lower TGF-beta1 expression in colon carcinoma: a retrospective study. BMC Cancer 7: 156. 17692120 10.1186/1471-2407-7-156 PMC1988827

[31] Ishigami S, Natsugoe S, Tokuda K, Nakajo A, Okumura H, et al (2003) Tumor-associated macrophage (TAM) infiltration in gastric cancer. Anticancer Res 23: 4079–4083. 14666722

[32] Haas M, Dimmler A, Hohenberger W, Grabenbauer GG, Niedobitek G, et al (2009) Stromal regulatory T-cells are associated with a favourable prognosis in gastric cancer of the cardia. BMC Gastroenterol 9: 65. 19732435 10.1186/1471-230X-9-65 PMC2749861

[33] Kawahara A, Hattori S, Akiba J, Nakashima K, Taira T, et al (2010) Infiltration of thymidine phosphorylase-positive macrophages is closely associated with tumor angiogenesis and survival in intestinal type gastric cancer. Oncol Rep 24: 405–415. 20596627 10.3892/or_00000873

[34] Osinsky S, Bubnovskaya L, Ganusevich I, Kovelskaya A, Gumenyuk L, et al (2011) Hypoxia, tumour-associated macrophages, microvessel density, VEGF and matrix metalloproteinases in human gastric cancer: Interaction and impact on survival. Clinical and Translational Oncology 13: 133–138. 21324802 10.1007/s12094-011-0630-0

[35] Ding T, Xu J, Wang F, Shi M, Zhang Y, et al (2009) High tumor-infiltrating macrophage density predicts poor prognosis in patients with primary hepatocellular carcinoma after resection. Hum Pathol 40: 381–389. 18992916 10.1016/j.humpath.2008.08.011

[36] Kuang DM, Zhao Q, Peng C, Xu J, Zhang JP, et al (2009) Activated monocytes in peritumoral stroma of hepatocellular carcinoma foster immune privilege and disease progression through PD-L1. J Exp Med 206: 1327–1337. 19451266 10.1084/jem.20082173 PMC2715058

[37] Kurahara H, Takao S, Kuwahata T, Nagai T, Ding Q, et al.. (2012) Clinical Significance of Folate Receptor beta-expressing Tumor-associated Macrophages in Pancreatic Cancer. Ann Surg Oncol. 10.1245/s10434-012-2263-0 22350599

[38] Hasita H, Komohara Y, Okabe H, Masuda T, Ohnishi K, et al (2010) Significance of alternatively activated macrophages in patients with intrahepatic cholangiocarcinoma. Cancer Sci 101: 1913–1919. 20545696 10.1111/j.1349-7006.2010.01614.x PMC11158749

[39] Leek RD, Lewis CE, Whitehouse R, Greenall M, Clarke J, et al (1996) Association of macrophage infiltration with angiogenesis and prognosis in invasive breast carcinoma. Cancer Res 56: 4625–4629. 8840975

[40] Toi M, Ueno T, Matsumoto H, Saji H, Funata N, et al (1999) Significance of thymidine phosphorylase as a marker of protumor monocytes in breast cancer. Clin Cancer Res 5: 1131–1137. 10353748

[41] Valkovic T, Dobrila F, Melato M, Sasso F, Rizzardi C, et al (2002) Correlation between vascular endothelial growth factor, angiogenesis, and tumor-associated macrophages in invasive ductal breast carcinoma. Virchows Arch 440: 583–588. 12070596 10.1007/s004280100458

[42] Bolat F, Kayaselcuk F, Nursal TZ, Yagmurdur MC, Bal N, et al (2006) Microvessel density, VEGF expression, and tumor-associated macrophages in breast tumors: correlations with prognostic parameters. J Exp Clin Cancer Res 25: 365–372. 17167977

[43] Mahmoud SM, Lee AH, Paish EC, Macmillan RD, Ellis IO, et al (2012) Tumour-infiltrating macrophages and clinical outcome in breast cancer. J Clin Pathol 65: 159–163. 22049225 10.1136/jclinpath-2011-200355

[44] Salvesen HB, Akslen LA (1999) Significance of tumour-associated macrophages, vascular endothelial growth factor and thrombospondin-1 expression for tumour angiogenesis and prognosis in endometrial carcinomas. Int J Cancer 84: 538–543. 10502735 10.1002/(sici)1097-0215(19991022)84:5<538::aid-ijc17>3.0.co;2-b

[45] Hashimoto I, Kodama J, Seki N, Hongo A, Miyagi Y, et al (2000) Macrophage infiltration and angiogenesis in endometrial cancer. Anticancer Res 20: 4853–4856. 11205231

[46] Soeda S, Nakamura N, Ozeki T, Nishiyama H, Hojo H, et al (2008) Tumor-associated macrophages correlate with vascular space invasion and myometrial invasion in endometrial carcinoma. Gynecol Oncol 109: 122–128. 18289648 10.1016/j.ygyno.2007.12.033

[47] Espinosa I, Jose Carnicer M, Catasus L, Canet B, D'Angelo E, et al (2010) Myometrial invasion and lymph node metastasis in endometrioid carcinomas: tumor-associated macrophages, microvessel density, and HIF1A have a crucial role. Am J Surg Pathol 34: 1708–1714. 20962622 10.1097/PAS.0b013e3181f32168

[48] Lissbrant IF, Stattin P, Wikstrom P, Damber JE, Egevad L, et al (2000) Tumor associated macrophages in human prostate cancer: relation to clinicopathological variables and survival. Int J Oncol 17: 445–451. 10938382 10.3892/ijo.17.3.445

[49] Shimura S, Yang G, Ebara S, Wheeler TM, Frolov A, et al (2000) Reduced infiltration of tumor-associated macrophages in human prostate cancer: association with cancer progression. Cancer Res 60: 5857–5861. 11059783

[50] Nonomura N, Takayama H, Nakayama M, Nakai Y, Kawashima A, et al (2011) Infiltration of tumour-associated macrophages in prostate biopsy specimens is predictive of disease progression after hormonal therapy for prostate cancer. BJU Int 107: 1918–1922. 21044246 10.1111/j.1464-410X.2010.09804.x

[51] Heller DS, Hameed M, Cracchiolo B, Wiederkehr M, Scott D, et al (2003) Presence and quantification of macrophages in squamous cell carcinoma of the cervix. Int J Gynecol Cancer 13: 67–70. 12631223 10.1046/j.1525-1438.2003.13035.x

[52] Kawanaka T, Kubo A, Ikushima H, Sano T, Takegawa Y, et al (2008) Prognostic significance of HIF-2alpha expression on tumor infiltrating macrophages in patients with uterine cervical cancer undergoing radiotherapy. J Med Invest 55: 78–86. 18319549 10.2152/jmi.55.78

[53] Hanada T, Nakagawa M, Emoto A, Nomura T, Nasu N, et al (2000) Prognostic value of tumor-associated macrophage count in human bladder cancer. Int J Urol 7: 263–269. 10910229 10.1046/j.1442-2042.2000.00190.x

[54] Takayama H, Nishimura K, Tsujimura A, Nakai Y, Nakayama M, et al (2009) Increased infiltration of tumor associated macrophages is associated with poor prognosis of bladder carcinoma in situ after intravesical bacillus Calmette-Guerin instillation. J Urol 181: 1894–1900. 19237175 10.1016/j.juro.2008.11.090

[55] Tanaka Y, Kobayashi H, Suzuki M, Kanayama N, Terao T (2004) Upregulation of bikunin in tumor-infiltrating macrophages as a factor of favorable prognosis in ovarian cancer. Gynecol Oncol 94: 725–734. 15350365 10.1016/j.ygyno.2004.06.012

[56] Takanami I, Takeuchi K, Kodaira S (1999) Tumor-associated macrophage infiltration in pulmonary adenocarcinoma: association with angiogenesis and poor prognosis. Oncology 57: 138–142. 10461061 10.1159/000012021

[57] Chen JJ, Lin YC, Yao PL, Yuan A, Chen HY, et al (2005) Tumor-associated macrophages: the double-edged sword in cancer progression. J Clin Oncol 23: 953–964. 15598976 10.1200/JCO.2005.12.172

[58] Zeni E, Mazzetti L, Miotto D, Lo Cascio N, Maestrelli P, et al (2007) Macrophage expression of interleukin-10 is a prognostic factor in nonsmall cell lung cancer. Eur Respir J 30: 627–632. 17537769 10.1183/09031936.00129306

[59] Al-Shibli Ibrahim K, Al-Saad S, Donnem T, Persson M, Bremnes R, et al (2009) The prognostic value of intraepithelial and stromal innate immune system cells in non-small cell lung carcinoma. Virchows Archiv 455: S85. 10.1111/j.1365-2559.2009.03379.x 19723145

[60] Ohtaki Y, Ishii G, Nagai K, Ashimine S, Kuwata T, et al (2010) Stromal macrophage expressing CD204 is associated with tumor aggressiveness in lung adenocarcinoma. J Thorac Oncol 5: 1507–1515. 20802348 10.1097/JTO.0b013e3181eba692

[61] Zhang BC, Gao J, Wang J, Rao ZG, Wang BC, et al (2011) Tumor-associated macrophages infiltration is associated with peritumoral lymphangiogenesis and poor prognosis in lung adenocarcinoma. Med Oncol 28: 1447–1452. 20676804 10.1007/s12032-010-9638-5

[62] Fujii N, Shomori K, Shiomi T, Nakabayashi M, Takeda C, et al.. (2012) Cancer-associated fibroblasts and CD163-positive macrophages in oral squamous cell carcinoma: their clinicopathological and prognostic significance. J Oral Pathol Med. 10.1111/j.1600-0714.2012.01127.x 22296275

[63] Lin JY, Li XY, Tadashi N, Dong P (2011) Clinical significance of tumor-associated macrophage infiltration in supraglottic laryngeal carcinoma. Chin J Cancer 30: 280–286. 21439250 10.5732/cjc.010.10336 PMC4013355

[64] Ryder M, Ghossein RA, Ricarte-Filho JC, Knauf JA, Fagin JA (2008) Increased density of tumor-associated macrophages is associated with decreased survival in advanced thyroid cancer. Endocr Relat Cancer 15: 1069–1074. 18719091 10.1677/ERC-08-0036 PMC2648614

[65] Burt BM, Rodig SJ, Tilleman TR, Elbardissi AW, Bueno R, et al (2011) Circulating and tumor-infiltrating myeloid cells predict survival in human pleural mesothelioma. Cancer 117: 5234–5244. 21523763 10.1002/cncr.26143

[66] Gordon S (2003) Alternative activation of macrophages. Nat Rev Immunol 3: 23–35. 12511873 10.1038/nri978

[67] Xie QW, Cho HJ, Calaycay J, Mumford RA, Swiderek KM, et al (1992) Cloning and characterization of inducible nitric oxide synthase from mouse macrophages. Science 256: 225–228. 1373522 10.1126/science.1373522

[68] Bonta IL, Ben-Efraim S (1993) Involvement of inflammatory mediators in macrophage antitumor activity. J Leukoc Biol 54: 613–626. 8245715 10.1002/jlb.54.6.613

[69] Nesbit M, Schaider H, Miller TH, Herlyn M (2001) Low-level monocyte chemoattractant protein-1 stimulation of monocytes leads to tumor formation in nontumorigenic melanoma cells. J Immunol 166: 6483–6490. 11359798 10.4049/jimmunol.166.11.6483

[70] Umemura N, Saio M, Suwa T, Kitoh Y, Bai J, et al (2008) Tumor-infiltrating myeloid-derived suppressor cells are pleiotropic-inflamed monocytes/macrophages that bear M1- and M2-type characteristics. J Leukoc Biol 83: 1136–1144. 18285406 10.1189/jlb.0907611

[71] Oosterling SJ, van der Bij GJ, Meijer GA, Tuk CW, van Garderen E, et al (2005) Macrophages direct tumour histology and clinical outcome in a colon cancer model. J Pathol 207: 147–155. 16104052 10.1002/path.1830

[72] Stewart LA, Parmar MK (1993) Meta-analysis of the literature or of individual patient data: is there a difference? Lancet 341: 418–422. 8094183 10.1016/0140-6736(93)93004-k

[73] Marcus B, Arenberg D, Lee J, Kleer C, Chepeha DB, et al (2004) Prognostic factors in oral cavity and oropharyngeal squamous cell carcinoma. Cancer 101: 2779–2787. 15546137 10.1002/cncr.20701

[74] Sickert D, Aust DE, Langer S, Haupt I, Baretton GB, et al (2005) Characterization of macrophage subpopulations in colon cancer using tissue microarrays. Histopathology 46: 515–521. 15842633 10.1111/j.1365-2559.2005.02129.x

[75] Liguori M, Solinas G, Germano G, Mantovani A, Allavena P (2011) Tumor-associated macrophages as incessant builders and destroyers of the cancer stroma. Cancers 3: 3740–3761. 24213109 10.3390/cancers3043740 PMC3763394

[76] Espinosa I, Edris B, Lee CH, Cheng HW, Gilks CB, et al (2011) CSF1 expression in nongynecological leiomyosarcoma is associated with increased tumor angiogenesis. Am J Pathol 179: 2100–2107. 21854753 10.1016/j.ajpath.2011.06.021 PMC3181338

[77] Valkovic T, Lucin K, Krstulja M, Dobi-Babic R, Jonjic N (1998) Expression of monocyte chemotactic protein-1 in human invasive ductal breast cancer. Pathol Res Pract 194: 335–340. 9651946 10.1016/S0344-0338(98)80057-5

[78] Ueno T, Toi M, Saji H, Muta M, Bando H, et al (2000) Significance of macrophage chemoattractant protein-1 in macrophage recruitment, angiogenesis, and survival in human breast cancer. Clin Cancer Res 6: 3282–3289. 10955814

[79] Fujimoto H, Sangai T, Ishii G, Ikehara A, Nagashima T, et al (2009) Stromal MCP-1 in mammary tumors induces tumor-associated macrophage infiltration and contributes to tumor progression. Int J Cancer 125: 1276–1284. 19479998 10.1002/ijc.24378

[80] Ohba K, Miyata Y, Kanda S, Koga S, Hayashi T, et al (2005) Expression of urokinase-type plasminogen activator, urokinase-type plasminogen activator receptor and plasminogen activator inhibitors in patients with renal cell carcinoma: correlation with tumor associated macrophage and prognosis. J Urol 174: 461–465. 16006865 10.1097/01.ju.0000165150.46006.92

[81] Avila-Moreno F, Lopez-Gonzalez JS, Galindo-Rodriguez G, Prado-Garcia H, Bajana S, et al (2006) Lung squamous cell carcinoma and adenocarcinoma cell lines use different mediators to induce comparable phenotypic and functional changes in human monocyte-derived dendritic cells. Cancer Immunol Immunother 55: 598–611. 16133109 10.1007/s00262-005-0060-3 PMC11029896

[82] Verreck FA, de Boer T, Langenberg DM, van der Zanden L, Ottenhoff TH (2006) Phenotypic and functional profiling of human proinflammatory type-1 and anti-inflammatory type-2 macrophages in response to microbial antigens and IFN-gamma- and CD40L-mediated costimulation. J Leukoc Biol 79: 285–293. 16330536 10.1189/jlb.0105015

[83] Wang R, Lu M, Zhang J, Chen S, Luo X, et al (2011) Increased IL-10 mRNA expression in tumor-associated macrophage correlated with late stage of lung cancer. J Exp Clin Cancer Res 30: 62. 21595995 10.1186/1756-9966-30-62 PMC3117740

[84] Simon R, Altman DG (1994) Statistical aspects of prognostic factor studies in oncology. Br J Cancer 69: 979–985. 8198989 10.1038/bjc.1994.192 PMC1969431

